# Seroincidence of Enteric Fever, Juba, South Sudan

**DOI:** 10.3201/eid2811.220239

**Published:** 2022-11

**Authors:** Kristen Aiemjoy, John Rumunu, Juma John Hassen, Kirsten E. Wiens, Denise Garrett, Polina Kamenskaya, Jason B. Harris, Andrew S. Azman, Peter Teunis, Jessica C. Seidman, Joseph F. Wamala, Jason R. Andrews, Richelle C. Charles

**Affiliations:** University of California Davis School of Medicine, Davis, California, USA (K. Aiemjoy);; Republic of South Sudan Ministry of Health, Juba, South Sudan (J. Rumunu);; World Health Organization, Juba (J.J. Hassen, J.F. Wamala);; Johns Hopkins Bloomberg School of Public Health, Baltimore, Maryland, USA (K.E. Wiens, A.S. Azman);; Sabin Vaccine Institute, Washington, DC, USA (D. Garrett, J.C. Seidman);; Massachusetts General Hospital, Boston, Massachusetts, USA (P. Kamenskaya, J.B. Harris, R.C. Charles);; Emory University, Atlanta, Georgia, USA (P. Teunis);; Stanford University School of Medicine, Stanford, California, USA (J.R. Andrews)

**Keywords:** enteric fever, Juba, South Sudan, enteric infections, bacteria, salmonella, typhoidal salmonella, Salmonella enterica, Typhi, Paratyphi

## Abstract

We applied a new serosurveillance tool to estimate typhoidal *Salmonella* burden using samples collected during 2020 from a population in Juba, South Sudan. By using dried blood spot testing, we found an enteric fever seroincidence rate of 30/100 person-years and cumulative incidence of 74% over a 4-year period.

Enteric fever, caused by *Salmonella enterica* serovars Typhi and Paratyphi, causes substantial illness and death globally (*1*). However, estimating the population-level burden of infection is challenging. Blood culture, the standard for both diagnosis and surveillance, requires microbiological laboratory facilities that are not available in many low- and-middle-income countries. Challenges in accessing blood culture, along with an estimated diagnostics sensitivity of only 60% (*2*), contribute to chronic underdetection (*3*).

Juba, the capital of South Sudan, experiences a high burden of enteric infections such as cholera and hepatitis E virus (*4*,*5*). Enteric fever is a frequently diagnosed etiology of acute fever, but laboratories have blood culture capacity for confirmation. Consequentially, the population-level burden of enteric fever is unknown.

Hemolysin E (HlyE), a pore-forming toxin, is a sensitive and specific serologic marker for diagnosing typhoidal *Salmonella* (*6*–*10*) and is not associated with typhoid carriage (*11*). New serologic and analytic tools enable measurement of population-level enteric fever incidence from cross-sectional serosurveys using HlyE IgG and IgA (*12*). We applied those tools to generate population-level enteric fever seroincidence estimates in Juba.

## The Study

We used dried blood spots (DBS) collected for a SARS-CoV-2 serosurvey in Juba, South Sudan, enrolled during August 7–September 20, 2020; enrollment and sampling methods are described elsewhere (*13*). In brief, 2-stage cluster sampling was used to randomly select households from predefined enumeration units from 6 administrative divisions within and surrounding Juba; all persons >1 year of age and residing for >1 week within the sampled household were eligible to participate. Capillary blood was collected onto Whatman 903 Protein Saver cards (Sigma-Aldrich, https://www.sigmaaldrich.com), air dried, and transported at ambient temperature to Massachusetts General Hospital (Boston, MA, USA), where they were stored at 4°C. We tested all banked samples collected from participants <25 years of age and a random sample of participants >25 years of age. Younger participants were prioritized because they matched the age distribution of typhoid case data used for the seroincidence estimation (*12*). The study protocol was approved by ethical review boards with the South Sudan Ministry of Health and Massachusetts General Hospital.

We used kinetic ELISAs to quantify HlyE IgA and IgG levels in eluted DBS as described (*7*,*11*). To estimate seroincidence, we used the antibody dynamics from a longitudinal cohort of 1,420 blood culture–confirmed enteric fever cases (*12*). In brief, we created a likelihood function for observed cross-sectional population antibody response data based on antibody dynamics after blood-culture confirmed infection. We generated joint incidence estimates by combining the likelihood for HlyE IgA and IgG for each age stratum using age-specific antibody dynamics. We selected age strata to match incidence estimates from blood culture enteric fever surveillance studies in other countries in sub-Saharan Africa and South Asia (*14*,*15*). This method incorporates heterogeneity in antibody responses and explicitly accounts for measurement error and biologic noise (*12*; Appendix reference *16*). 

We used 3 US populations to define the distribution of biologic noise (nonspecific antibody binding): 48 children 1–5 years of age who had relatives with celiac disease, enrolled nationwide; 31 healthy controls, children and young adults 2–18 years of age, enrolled at Massachusetts General Hospital (Appendix reference *17*); and a population-based sample of 205 children and adults 3–50 years of age from a SARS-CoV-2 serosurvey in northern California, USA. We used the same method to generate individual-level incidence estimates of HlyE IgA and IgG responses and used the exponential probability distribution to calculate 2- and 4-year cumulative incidence. We then fit age-dependent curves by using generalized additive models (Appendix reference *18*) with a cubic spline for age and simultaneous 95% CIs using a parametric bootstrap of the variance-covariance matrix of the fitted model parameters (Appendix reference *19*).

A total of 2,214 persons were enrolled and provided blood samples for the original study; 1,840 had complete interview data, and 1,290 were randomly selected for testing (*13*). The median age of tested participants was 17 (interquartile range [IQR] 10–24) years; 63.5% (819/1,290) were female (Table 1).

**Table 1:** Demographic characteristics of participants in study of seroincidence of enteric fever, Juba, South Sudan, 2020*

Characteristic	Value, N = 1,290
Sex	
F	819 (63.5)
M	471 (36.5)
Age, y, median (IQR)	17 (10–24)
Age category, in years	
1–3	41 (3.2)
4–6	118 (9.1)
7–9	134 (10.4)
10–14	259 (20.1)
15–24	423 (32.8)
25–34	167 (12.9)
35–44	62 (4.8)
>45	86 (6.7)

*Values are no. (%) except as indicated. IQR, interquartile range.

**Figure 1:**
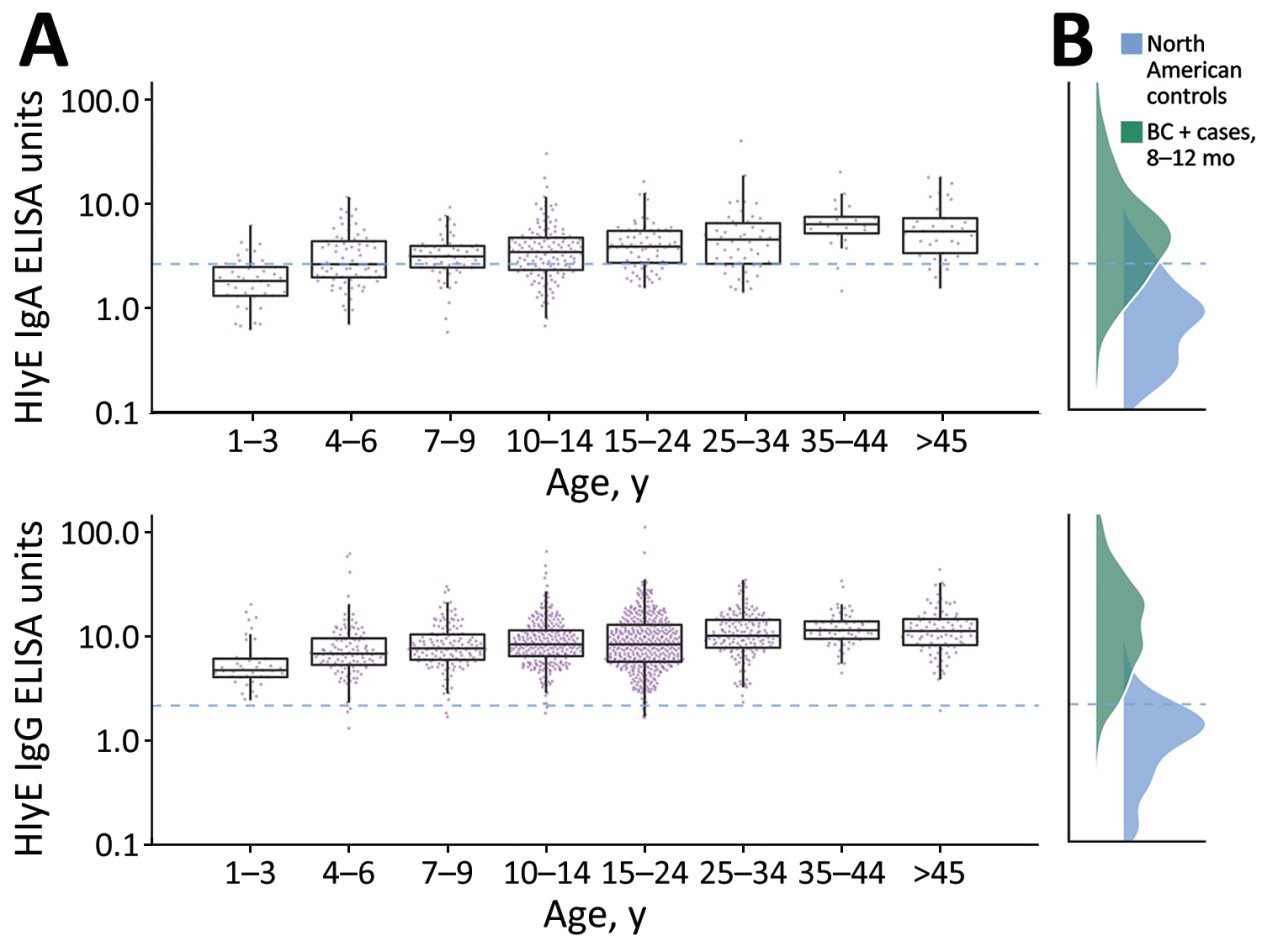
Age-dependent hemolysin E (HlyE) IgA (top) and IgG (bottom) responses for participants in study of seroincidence of enteric fever, Juba, South Sudan, 2020, compared with those for blood culture-confirmed cases and controls. A) Cross-sectional antibody responses to HlyE IgA (top) and IgG (bottom) by age measured from a serosurvey of 1,290 persons in Juba, South Sudan, from samples during collected during August 7–September 2, 2020. Each point indicates an individual sample. Horizontal lines within boxes indicate medians; box tops and bottoms indicate IQRs; error bars indicate 95% CIs. B) Density of antibody responses HlyE IgA (top) and IgG (bottom) among 1,410 blood-culture confirmed enteric fever cases in Bangladesh, Nepal, Pakistan, and Ghana 8–12 months after symptom onset as reported in (*12*) and a control population from 3 United States groups: 48 children 1–5 years of age who had first degree relatives with celiac disease, enrolled nationally; 31 healthy controls, children and young adults 2–18 years of age, enrolled at Massachusetts General Hospital (Appendix reference *17*); and a population-based sample of 205 children and adults 3–50 years of age participating in a SARS-CoV serosurvey in California, USA. The dashed blue line across all panels represents the mean +3 SD of HlyE IgA and IgG values observed in the pediatric control population. HlyE, hemolysin E.

**Figure 2:**
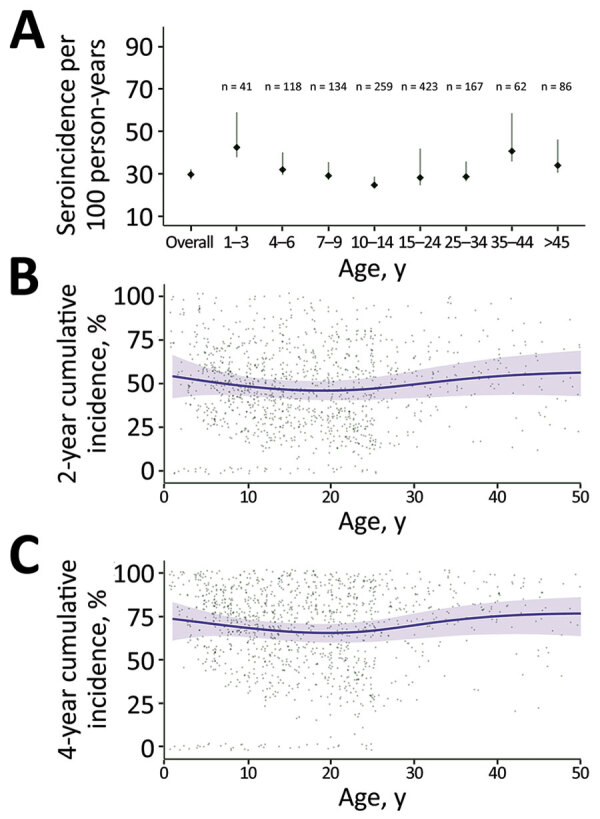
Estimated seroincidence of typhoidal *Salmonella* by age, Juba, South Sudan, 2020. A) Seroincidence per age group. Error bars indicate 95% CIs. B, C) Individually predicted incidence estimates (points) and smoothed cumulative incidence (lines) over 2-year (B) and 4-year (C) periods, by age. Gray shading indicates 95% CIs.

We found that median HlyE IgG (10.4, IQR 6.1–12.7) and IgA (3.5, IQR 2.3–5.2) responses were elevated well above a North America pediatric control population (IgG 0.16, IQR 0.07–0.35; IgA 0.3, IQR 0.001–0.92) and were comparable to responses observed among blood-culture confirmed enteric fever cases 8–12 months after symptom onset (IgG 12, IQR 5.9–24; IgA 4.4, IQR 2.2–9.4) (*12*) (Figure 1). Age-specific enteric fever incidence estimates per 100 person-years ranged from 24.8 (95% CI 23.6–28.8) among children 10–14 years of age to 42.5 (95% CI 38.0–59.0) among children 1–3 years of age (Table 2; Figure 2). The overall incidence rate was 29.8 (95% CI 27.6–32.2); cumulative incidence was 48.9% (IQR 31.9–64.3) over 2 years and 73.8% (IQR 53.7–87.3) over 4 years. Using a cutoff derived from a North America pediatric control population, we found 98.8% (1,275/1,290) of the population seropositive using HlyE IgG and 65.2% (318/488) positive using HlyE IgA (Appendix).

**Table 2:** Age-dependent incidence rates and cumulative incidence for participants in study of seroincidence of enteric fever, Juba, South Sudan, 2020*

Age group, y	Seroincidence, cases/100 person-years (95% CI)	2-year cumulative incidence, % (IQR)	4-year cumulative incidence, % (IQR)
1–3	42.5 (38.0–59.0)	53.6 (44.5–74.9)	78.5 (69.2–93.7)
4–6	32.1 (29.7–40.1)	56.6 (34.7–68.5)	81.2 (57.4–90.0)
7–9	29.2 (27.3–35.6)	49.3 (39.9–59.1)	74.3 (63.8–83.3)
10–14	24.8 (23.6–28.8)	45.1 (30.9–58.4)	69.9 (52.2–82.7)
15–24	28.3 (24.7–41.9)	42.8 (27.2–61.6)	67.3 (47.0–85.3)
25–34	28.8 (26.7–35.9)	51.7 (35.7–66.5)	76.7 (58.6–88.8)
35–44	40.8 (36.0–58.5)	57.5 (49.8–69.5)	82.0 (74.8–90.7)
>45	34.0 (30.6–46.2)	53.0 (40.9–69.1)	77.9 (65.1–90.5)
Overall	29.8 (27.6–32.2)	48.9 (31.9–64.3)	73.8 (53.7–87.3)

*IQR, interquartile range.

## Conclusions

Using banked DBS collected for a SARS-CoV-2 serosurvey, we applied a new serosurveillance tool to rapidly estimate the burden of enteric fever in a region with no blood culture surveillance. We estimated an incidence rate of 30.0 infections/100 person-years and found >70% of the sampled population was infected in the previous 4 years.

Whereas no clinical enteric fever incidence estimates from South Sudan are available for comparison, the seroincidence rate we estimated is substantially higher than clinical incidence estimates in the region (*15*; Appendix reference *20*). A high incidence of clinical enteric fever has been previously defined as >100 cases/100,000 person-years (Appendix reference *21*); we estimated a seroincidence of 35,000 cases/100,000 person-years. We expect seroincidence to be higher than clinical incidence because it captures subclinical infections and is independent of a person’s ability to access and afford healthcare, including diagnostic tests. Indeed, the enteric fever seroincidence rate for Juba is on the same scale of magnitude as recent estimates using the same approach in Nepal, Pakistan, Bangladesh, and Ghana (*12*).

The analytic approach is an improvement over cutoff-based methods because we can combine information from HlyE IgA and IgG responses to generate a consensus incidence estimate, accounting for heterogeneity in antibody responses, measurement error, and biologic noise. Whereas the cutoff-based method yielded a seroprevalence of nearly 100% for HlyE IgG, we generated cumulative incidence estimates over a precise time window and could identify populations with recent and later infections.

Limitations of this study include that only 1,840 samples of 2,214 enrolled study participants had linked age data. Second, persons in internally displaced camps were not included in the serosurvey. Displaced persons have been identified as high-risk populations for enteric infections, so it would be valuable to include them in future studies to determine if this population is at higher or equivalent risk (*4*). Finally, we used longitudinal antibody kinetics estimates from enteric fever cases in Bangladesh, Pakistan, Nepal, and Ghana. We did not observe major differences in the kinetics of antibody responses across countries (*12*), but the decay rate among enteric fever cases in Juba may be different because of the high force of infection and differences in exposure to other infections.

Our results suggest a high burden of enteric fever in Juba, South Sudan, warranting urgent public health and research attention. The seroincidence tool we used can be applied to other regions lacking blood culture surveillance to generate rapid enteric fever seroincidence estimates, providing the high-resolution data critically needed to inform typhoid conjugate vaccine introduction.

Appendix Additional information for study of seroincidence of enteric fever, Juba, South Sudan.
