# Supplementary material for: Seroincidence of Enteric Fever, Juba, South Sudan
Source: Emerg Infect Dis. 2022 Nov;28(11):2316–20. doi: 10.3201/eid2811.220239 (PMC9622235; doi:10.3201/eid2811.220239)
Supplement: Appendix — Additional information for study of seroincidence of enteric fever, Juba, South Sudan. [file 22-0239-Techapp-s1.pdf]

Article DOI: http://doi.org/10.3201/eid2811.220239

Seroincidence of Enteric Fever, Juba, South Sudan

Appendix

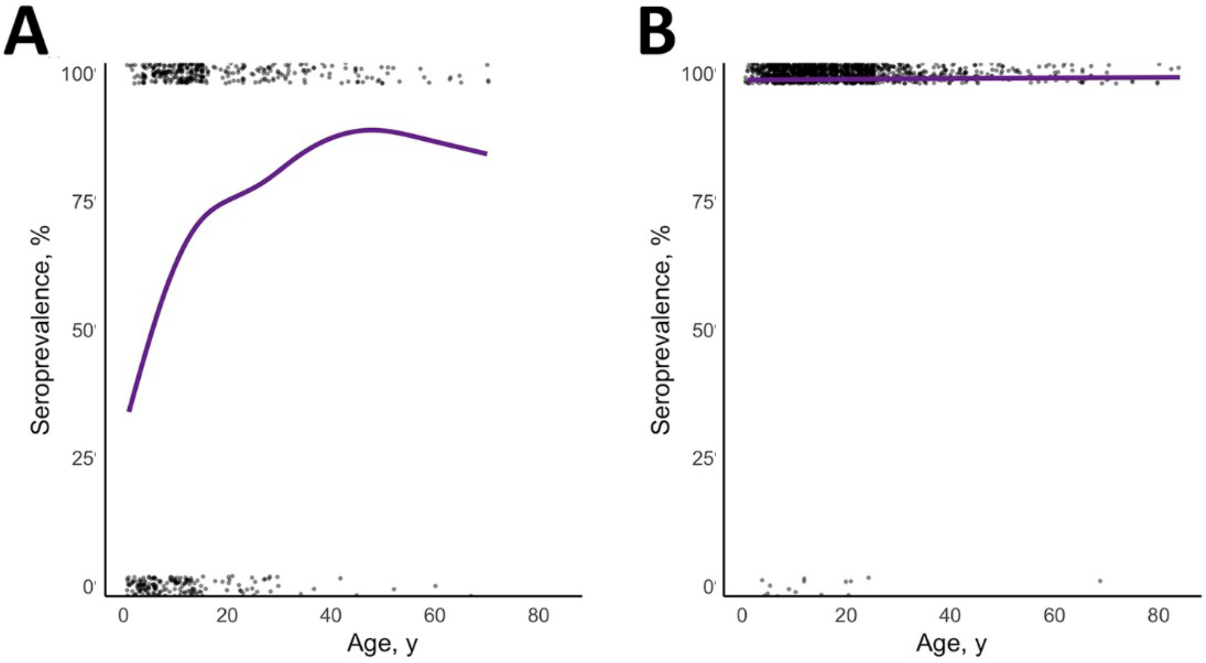

Appendix Figure. Age-dependent seroprevalence curves using negative control cutoff. Each point is an individual classified into positive or negative based on a cutoff derived from a North American control population for hemolysin E immunoglobulin A (HlyE IgA) (left) and hemolysin E immunoglobulin G (HlyE IgG) (right). The purple line is the age-dependent seroprevalence curve fit using generalized additive models.

Additional References

16. Teunis PFM, van Eijkeren JCH. Estimation of seroconversion rates for infectious diseases: effects of age and noise. Stat Med. 2020;39:2799–814. PubMed https://doi.org/10.1002/sim.8578

17. Leonard MM, Camhi S, Huedo-Medina TB, Fasano A. Celiac Disease Genomic, Environmental, Microbiome, and Metabolomic (CDGEMM) study design: approach to the future of personalized prevention of celiac disease. Nutrients. 2015;7:9325–36. PubMed https://doi.org/10.3390/nu7115470

18. Wood SN. Generalized additive models: an introduction with R. London: Chapman and Hall/CRC; 2006.

19. Marra G, Wood SN. Coverage properties of confidence intervals for generalized additive model components. Scand J Stat. 2012;39:53–74. https://doi.org/10.1111/j.1467-9469.2011.00760.x

20. Meiring JE, Shakya M, Khanam F, Voysey M, Phillips MT, Tonks S, et al.; STRATAA Study Group. Burden of enteric fever at three urban sites in Africa and Asia: a multicentre population-based study. Lancet Glob Health. 2021;9:e1688–96. PubMed https://doi.org/10.1016/S2214-109X(21)00370-3

21. Crump JA, Luby SP, Mintz ED. The global burden of typhoid fever. Bull World Health Organ. 2004;82:346–53. PubMed
